# *PD-1* and *PD-L1* Expression Levels as a Potential Biomarker of Chronic Rhinosinusitis and Head and Neck Cancers

**DOI:** 10.3390/jcm12052033

**Published:** 2023-03-03

**Authors:** Katarzyna Malinowska, Andrzej Kowalski, Anna Merecz-Sadowska, Milena Paprocka-Zjawiona, Przemysław Sitarek, Tomasz Kowalczyk, Hanna Zielińska-Bliźniewska

**Affiliations:** 1 Department of Allergology and Respiratory Rehabilitation, Medical University of Lodz, 90-725 Lodz, Poland; 2 Department of Otolaryngology, Laryngological Oncology, Audiology and Phoniatrics, Medical University of Lodz, 90-549 Lodz, Poland; 3 Department of Biology and Pharmaceutical Botany, Medical University of Lodz, 90-151 Lodz, Poland; 4 Department of Molecular Biotechnology and Genetics, University of Lodz, 90-237 Lodz, Poland

**Keywords:** chronic rhinosinusitis with nasal polyps, head and neck cancer, inflammation, *PD-1*, *PD-L1*

## Abstract

Inflammation is an etiological factor of various chronic diseases contributing to more than 50% of worldwide deaths. In this study, we focus on the immunosuppressive role of the programmed death-1 (PD-1) receptor and its ligand (PD-L1) in inflammatory-related diseases, including chronic rhinosinusitis and head and neck cancers. The study included 304 participants. Of this number, 162 patients had chronic rhinosinusitis with nasal polyps (CRSwNP), 40 patients had head and neck cancer (HNC) and there were 102 healthy subjects. The expression level of the *PD-1* and *PD-L1* genes in the tissues of the study groups was measured by qPCR and Western blot methods. The associations between the age of the patients and the extent of disease and genes’ expression were evaluated. The study showed a significantly higher mRNA expression of *PD-1* and *PD-L1* in the tissues of both the CRSwNP and HNC patient groups compared to the healthy group. The severity of CRSwNP significantly correlated with the mRNA expression of *PD-1* and *PD-L1*. Similarly, the age of the NHC patients influenced *PD-L1* expression. In addition, a significantly higher level of PD-L1 protein was noticed also for both the CRSwNP and HNC patient groups. The increased expression of *PD-1* and *PD-L1* may be a potential biomarker of inflammatory-related diseases, including chronic rhinosinusitis and head and neck cancers.

## 1. Introduction

According to statistics, more than 50% of worldwide deaths are related to inflammatory diseases. Inflammation is an important process involving the induction of various cells and mediators. That mechanism protects the human body against numerous harmful biological, chemical or physical stimuli. Normally, inflammatory response is characterized by the temporal upregulation of inflammatory activity that resolves after dangers have subsided and is followed by the restoration of cellular homeostasis. However, inflammatory response shifts from short-lived to chronic may lead to alterations in all tissues. Furthermore, impaired immune response increases susceptibility to infection and even tumor. There are numerous inflammation-related disorders, and they include chronic rhinosinusitis but also head and neck cancers [1,2,3,4].

Rhinosinusitis (RS) is defined as inflammation of the mucosal membranes in the paranasal sinuses and nasal cavity. According to the European Position Paper on Rhinosinusitis and Nasal Polyps (EPOS), published in 2020, RS is classified into acute rhinosinusitis (ARS) and chronic rhinosinusitis (CRS) [5]. CRS is characterized by the presence of at least two out of four cardinal symptoms (i.e., facial pain/pressure, hyposmia/anosmia, nasal drainage and nasal obstruction) for at least 12 consecutive weeks, in addition to objective evidence [6]. A general population-based study indicates that CRS prevalence ranges from 3.0% to 6.4% [7]. The main risk factors for CRS include genetic, environmental and demographic factors, as well as the coexistence of diseases such as airway, inflammatory and autoimmune diseases. Recent studies show that the genes responsible for antigen presentation, innate and adaptive immune responses, tissue remodeling and arachidonic acid metabolism may be crucial in the pathogenesis of CRS [8,9,10]. CRS is divided into two subgroups: CRS with nasal polyps (CRSwNP) and CRS without nasal polyps (CRSsNP) [11]. According to the literature, the prevalence of CRSwNP ranges from 1% to 2.6% [12]. CRSwNP patients are characterized by a more pronounced nasal obstruction and progressive loss of smell than CRSsNP patients [13]. CRSwNP is most often characterized by type 2 inflammation [14]. However, distinguishing between two types of CRS can be difficult if we exclude nasal endoscopy [15]. 

A population-based study has demonstrated an association between CRS and a higher incidence of head and neck cancers [16]. Head and neck cancer (HNC) is a frequent malignancy involving the upper respiratory or digestive tract—lips, oral cavity, oropharynx, nasal cavity, nasopharynx, hypopharynx and larynx and upper trachea, as well as sinonasal cavities and salivary glands. Annually, approximately 900,000 new cases of HNCs are detected, and the disease contributes to 400,000 deaths worldwide. The main risks factors are tobacco consumption, alcohol abuse and human papillomavirus (HPV) infection. According to the professional literature, the pathogenesis of HNC is strictly associated with inflammation and elevated levels of pro-inflammatory factors. However, immune response as well as the presence of tumor-infiltrating lymphocytes (TILs) are a promising HNC biomarker. Improved outcomes are related with higher levels of T cells in the tumor microenvironment [17,18,19,20].

Programmed death-1 (PD-1) immunoreceptor was first described by Ishida Y. et al. [21]. The *PDC1* gene encoding the PD-1 protein is localized on the long arm of the 2q37 chromosome [22]. Primarily, it was found that the PD-1 receptor is placed on the hemopoietic cell surface. Later data revealed the inducible expression of *PD-1* on T and B cells, natural killer (NK) cells, macrophages and some dendritic cell (DC) subsets [23]. According to reports in the literature, the PD-1 pathway is widely analyzed in relation to immune disease [24] and cancer [25]. It was found that PD-1 is a negative regulator of immune response [26]. PD-1 binds to its ligands, PDL-L1 or PD-L2, and may inhibit positive cell activation, proliferation, cytokine secretion and survival [27]. Furthermore, there might be a significant relationship between the expression of *PD-1* or *PDL-1* and its possible involvement in inflammatory process [28,29,30,31]. The PD-1/PD-L1 axis is a target for monoclonal antibody treatments that have demonstrated some potential, but more studies are required to determine the beneficiaries of this therapy.

Previous literature investigating *PD-1* and *PD-L1* gene expression in tumor tissue from NHC patients showed higher levels of the mRNA and protein of both genes in subjects compared to the control, which was summarized by Lenouvel et al. [32]. There are currently limited data on the level of *PD-1* and *PD-L1* gene expression in polyp tissue. Considering that NHC and CRSwNP are related with inflammation and that CRS may be a risk factor of NHC, we hypothesized that a similar expression pattern may be characteristic for polyp tissue from CRSwNP patients. Therefore, the aim of this study was to analyze the expression patterns of *PD-1* and *PD-L1* genes in tissues collected from patients with CRSwNP and HNC and correlations between expression and clinical data.

## 2. Materials and Methods

### 2.1. Ethical Considerations 

The study was approved by the Bioethics Commission at the Medical University of Lodz (protocol no. RNN/140/15/KE; 19 May 2015). The study was performed in accordance with the ethical principles of the 1964 Helsinki Declaration. All participants were informed about the study and provided written informed consent before being recruited.

### 2.2. Patients

The study included 304 participants aged 18 years and above: 91 females and 213 males. These were divided into three groups, as follows:

I. Control group—102 healthy subjects with deviations of the nasal septum (DSN) with no features of cancers, chronic paranasal sinusitis or inflammatory diseases;
II. Study group—162 patients with CRSwNP;
III. Study group—40 patients with HNC.

The participants were hospitalized in the Department of Otolaryngology, Laryngological Oncology, Audiology and Phoniatrics of the Medical University Hospital in Lodz between 2021 and 2022. All patients and control subjects included in the study were Caucasian. The patients with CRSwNP underwent functional endoscopic sinus surgery (FESS). They did not use systemic corticosteroids and did not use antihistamines and topical corticosteroids for a period of one month prior to FESS. The patients with CRSwNP were diagnosed according to the EPOS 2020 criteria [5]. In order to analyze the severity of the disease, computed tomography (CT) scans were assessed using Kennedy scores. The staging system proposed by Kennedy divides changes throughout the sinus into four stages. Stage I involves patients with anatomic abnormalities, all unilateral sinus diseases and bilateral disease restricted to ethmoidal sinuses; stage II—bilateral ethmoidal disease with the involvement of one dependent sinus; and stage III—bilateral ethmoidal disease with involvement of two or more dependent sinuses on each side; stage IV—diffuse sinonasal polyposis [33]. Patients with HNC underwent surgical resection of the tumors. Tumors were assessed by histopathological examination. The tumor–node–metastasis (TNM) classification of cancer extent was used. The TNM staging system for solid tumors is based on tumor characteristics (T), nodal spread (N) and distant metastasis (M). The main purpose of the TNM staging system is to provide an anatomically based classification to depict cancer prognosis [34,35]. The participants’ characteristics are presented in detail in Table 1.

### 2.3. Materials

The following tissue samples were obtained from each study group:

Group I—fragment of the mucosa from the lower nasal concha, collected during endoscopic septoplasty;

Group II—polyp tissue of paranasal sinus collected during FESS;

Group III—tumor tissue collected during surgical procedures.

Immediately after collection, the tissue fragments were placed in Eppendorf tubes with 1 mL of RNAlater fluid (Qiagen, Hilden, Germany), preventing RNA degradation, and frozen at −20 °C. The expressions of the *PD-1* and *PD-L1* genes were evaluated in the materials.

### 2.4. Methods

#### 2.4.1. Total RNA Extraction and cDNA Generation

Total RNA extractions were carried out using RNeasy Mini Kit (Qiagen, Hilden, Germany) according to the manufacturer’s instructions. The RNA content and purity were measured using a PicoDrop spectrophotometer (Picodrop Limited, Hinxton, UK). The quality of the RNA samples was analyzed by measuring the ratio of absorptions at 260/280 nm. The purified total RNA was immediately used for cDNA synthesis or stored at −80 °C.

cDNA was generated with a Maxima First Strand cDNA Synthesis Kit (Thermo Fisher Scientific, Waltham, MA, USA) according to the manufacturer’s protocol. One milligram of total RNA was used as starting material; reverse transcription was performed in conditions optimized for use with this kit (25 °C for 10 min, 50 °C for 30 min and 85 °C for 5 min). The cDNA samples were kept frozen at −20 °C.

#### 2.4.2. Real-Time PCR

The TaqMan gene expression experiments were performed in 10 μL reactions including 50 ng cDNA, 5 μL TaqMan Universal PCR Master Mix and 0.5 μL appropriate TaqMan Gene Expression Assay (Thermo Fisher Scientific, Waltham, MA, USA). The specific TaqMan assays used in this study were programmed cell death 1 (PDCD1, Hs01550088_m1), programmed cell death 1 ligand 1 (PDCD1LG1, Hs01125301_m1) and β-actin (ACTB, Hs01060665_g1) as the endogenous control. The TaqMan PCR assays were performed on a 7900HT Fast Real-Time PCR System (Thermo Fisher Scientific, Waltham, MA, USA) in FastGene Fast 96-well PCR plates (Nippon Genetics Europe GmbH, Düren, Germany). The following thermal cycling specifications were performed: 20 s at 95 °C and 40 cycles each for 3 s at 95 °C and 30 s at 60 °C. The ΔΔCT method was used [36]. 

#### 2.4.3. Western Blot Analysis

Fifty milligrams of proteins were resolved on SDS electrophoresis gels with the use of standard procedures [37]. Immunoblotting was performed with anti-PD-1 rabbit antibody, PRS4065 (Merck, Darmstadt, Germany) or with anti-PD-L1 rabbit antibody, PRS4059 (Merck, Darmstadt, Germany). Final detection was achieved with the appropriate secondary antibody coupled to horseradish peroxidase (HRP), A0545 and anti-rabbit IgG-HRP (Merck, Darmstadt, Germany), followed by luminol-based chemiluminescent substrate (Cyanagen, Bologna, Italy) and visualized by a gel imaging system for chemiluminescence G: Box Chemi XR5 (Syngene, Cambridge, UK).

Equal loading of the proteins was additionally checked by the immunodetection of glyceraldehyde-3-phosphate dehydrogenase (GAPDH) mouse monoclonal antibody, MAB 374 (Merck Millipore, Burlington, MA, USA) and anti-mouse IgG-HRP, with A4416 as a secondary antibody (Sigma-Aldrich, Saint Louis, MO, USA), and visualized by G: Box Chemi XR5 (Syngene, Cambridge, UK). For the analysis of the protein band density, ImageJ 1.53k software (Wayne Rasband, USA) was used.

### 2.5. Statistics

The results are expressed as mean values ± standard deviation (SD). 

The differences between the samples were tested using one-way analysis of variance (ANOVA) with Tukey’s post hoc test after confirming the normality of the distribution and the homogeneity of variances. The normality of distribution was appraised using the Shapiro–Wilk test. The homogeneity of the variances was tested using Levene’s test. 

The Spearman correlation coefficient (r) was estimated to determine the linear association between the following variables: patient’s age and mRNA expression of the *PD-1* and *PD-L1* genes, extent of the disease and the mRNA expression of the *PD-1* and *PD-L1* genes. The outcome results were interpreted according to the degree of association as strong (r = 0.7–1), moderate (r = 0.5–0.7), or low (r = 0.3–0.5) after taking significant correlation (*p* < 0.05) values into consideration. 

A multiple linear regression model was implemented to predict the values of the dependent variables (i.e., mRNA expression level of *PD-1* and *PD-L1* genes) starting from knowledge of the independent variables (i.e., age and extent of the diseases). The equation for a multiple linear regression is:

y = β_0_ + β_1_x_1_ + β_2_x_2_ + ϵ

where y is the mRNA expression level of the *PD-1* and *PD-L1* genes, β_0_ is the intercept value, x_i_ are two independent variables, β_i_ are the estimated regression coefficients of the respective independent variables, and ε is the model error, i.e., the variation in our prediction of y with respect to the real value.

A level of *p* < 0.05 was considered statistically significant. All statistical computations were carried out in Statistica 13.0 software (StatSoft Polska, Cracow, Poland).

## 3. Results

### 3.1. mRNA Expression Level of the PD-1 and PD-L1 Genes in Tissues from CRSwNP and NHC Patients

**Figure 1:**
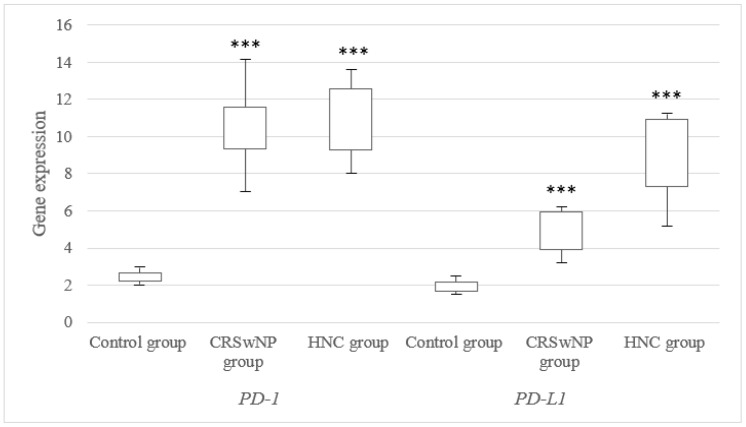
mRNA expression level of the PD-1 and PD-L1 genes in the examined patients with CRSwNP and HNC. *** *p* < 0.001 versus control group.

There were significant differences in the PD-1 and PD-L1 (*p* < 0.001) mRNA expression levels between polyp and normal tissues, with a 4.2-fold and 2.3-fold increase in the polyp tissues, respectively. Moreover, significant differences in the PD-1 and PD-L1 (*p* < 0.01) mRNA expression levels between tumor and normal tissues, with a 4.5-fold and 4.6-fold increase in the tumor tissues, were found, respectively. These data are presented in Figure 1.

### 3.2. mRNA Expression Level of the PD-1 and PD-L1 Genes in the Different Subgroups of CRSwNP and HNC Patients

The *PD-1* and *PD-L1* gene expression was evaluated in the different subgroups of the CRSwNP patients, including subjects with chronic rhinosinusitis with nasal polyps, chronic rhinosinusitis with nasal polyp and allergy and chronic rhinosinusitis with nonsteroidal anti-inflammatory drug (NSAID)-exacerbated respiratory disease (N-ERD). In addition, *PD-1* and *PD-L1* gene expression in nonsmoking and smoking groups of HNC patients was also assessed. The data is presented in Table 2.

In patients with CRSwNP and coexisting allergy, the mRNA level of *PD-1* in the polyp tissue was significantly elevated in comparison to that observed in the polyp tissue collected from patients with CRSwNP alone (*p* < 0.001). 

In smoking patients with HNC, the mRNA level of *PD-L1* in the tumor tissue was significantly elevated in comparison to that observed in tumor tissue collected from nonsmoking HNC patients (*p* = 0.037).

### 3.3. Correlation between the mRNA Expression Level of the PD-1 and PD-L1 Genes and the Ages of the CRSwNP and HNC Patients

**Figure 2:**
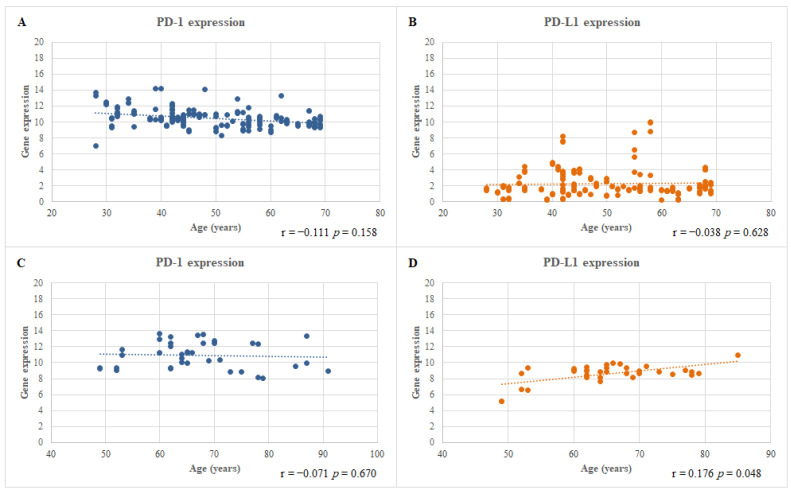
Correlation between the mRNA expression level of PD-1 (**A**,**C**) and PD-L1 (**B**,**D**) genes and the ages of CRSwNP patients (**A**,**B**) and of HNC patients (**C**,**D**).

In patients with CRSwNP, the mRNA levels of both the *PD-1* and *PD-L1* genes in the polyp tissue were not correlated with the disease severity calculated using the Kennedy scores. Conversely, in patients with HNC, the mRNA level of the *PD-L1* gene in the tumor tissue were correlated with the age of patients (*p* = 0.048). These data are presented in Figure 2.

### 3.4. Correlation between the mRNA Expression Level of the PD-1 and PD-L1 Genes and the Severity of the CRSwNP and HNC Diseases

**Figure 3:**
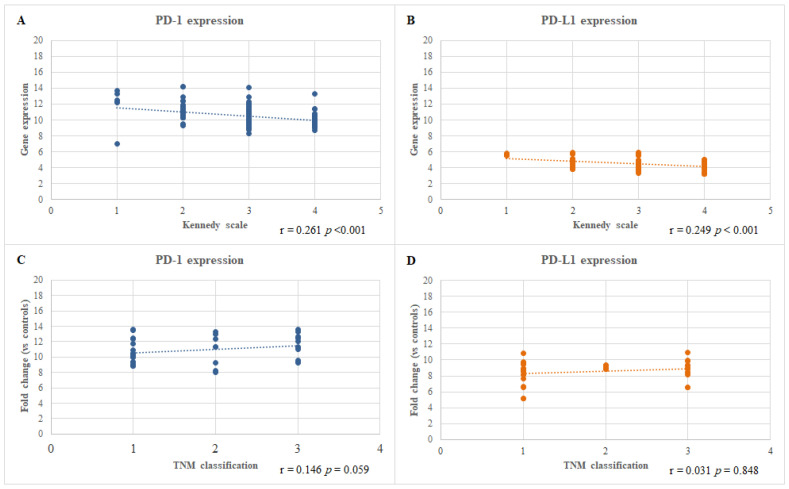
Correlation between the mRNA expression levels of the PD-1 (**A**,**C**) and PD-L1 (**B**,**D**) genes and the extent of the diseases calculated using the Kennedy score for the CRSwNP patients (**A**,**B**) and TNM staging system for the HNC patients (**C**,**D**).

In patients with CRSwNP, the mRNA levels of both the *PD-1* and *PD-L1* genes (*p* < 0.001) in the polyp tissue were correlated with the disease severity calculated using the Kennedy scores. Conversely, a similar correlation was not observed for patients with HNC calculated using the TNM staging system. These data are presented in Figure 3.

### 3.5. Multiple Linear Regression Analyses of PD-1 and PD-L1 mRNA Expression Levels

Table 3 shows the multiple linear regression analysis. According to the model investigated, a variable Kennedy scale demonstrated a significant contribution to the mRNA expression level of the *PD-L1* gene in the polyp tissue from CRSwNP patients. Moreover, the variable age demonstrated a significant contribution to the mRNA expression level of the *PD-L1* gene in the tumor tissue from HNC patients.

### 3.6. PD-1 and PD-L1 Protein Expression Level Analysis

**Figure 4:**
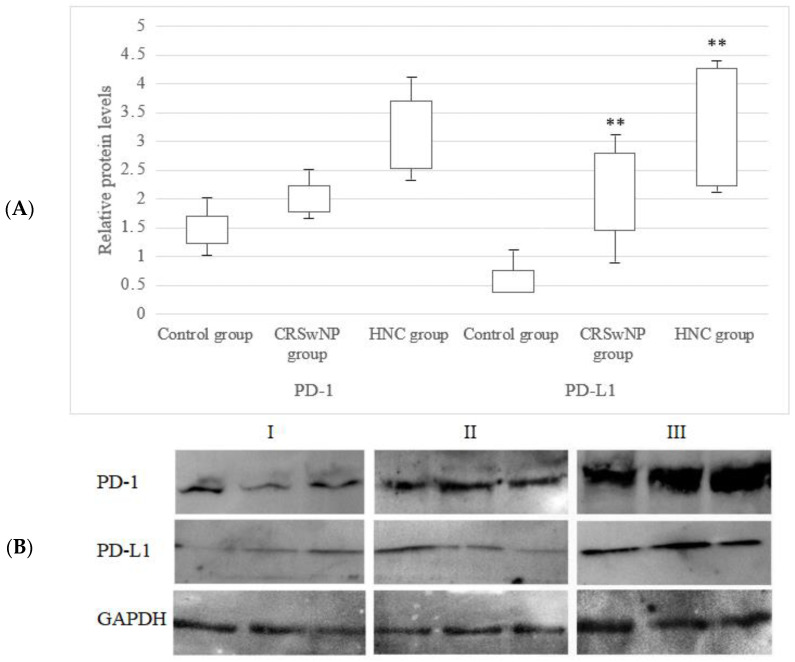
PD-1 and PD-L1 protein expressions in the tissue of the examined patients with CRSwNP and HNC (**A**). Representative Western blots of the PD-1 and PD-L1 protein expressions (**B**): control subjects (**I**); CRSwNP patients (**II**); HNC patients (**III**). ** *p* < 0.01 versus the control group.

An increased level of PD-L1 protein in the tissues from CRSwNP and HNC patients in comparison to the control subjects was confirmed. These data are presented in Figure 4.

Significant differences in the PD-L1 (*p* < 0.01) protein level between the polyp and normal tissues, with a 4.9-fold increase in the polyp tissues, were observed. Moreover, significant differences in the PD-L1 (*p* < 00.1) protein level between tumor and normal tissues, with a 5.7-fold increase in the tumor tissues, were found. The PD-1 protein level in both polyp and cancer tissues was also slightly increased in comparison to the control tissue, but the increase was not statistically significant.

## 4. Discussion

In our study, we investigated *PD-1* and *PD-L1* expression patterns in the tissues of CRSwNP patients. The data show increased mRNA and protein levels of *PD-L1* in polyp tissues from patients with CRSwNP compared to the control group. The expression of *PD-1* and its ligand *PD-L1* in CRSwNP patients is poorly investigated. It is indicated that *PD-1* expression is elevated in tissue infiltrating the T cells of CRSwNP patients [38]. Shi et al. have shown that the mRNA level of *PD-L1* significantly was increased in eosinophilic CRSwNP [39]. Furthermore, Krohn et al. have demonstrated an increased mRNA expression of *PD-1* in tissue homogenates from patients with CRSwNP compared to controls. Moreover, the mRNA level was associated with disease severity calculated using Lund-Mackay scores on CT scans [40]. According to our data, the severity of the CRSwNP calculated using Kennedy scores of CT scans was significantly correlated with the expression of both the *PD-1* and *PD-L1* genes.

The PD-1/PD-L1 axis was mainly explored in cancer cells. Various signaling pathways upregulate *PD-1/PD-L1* expression, including the phosphoinositide 3-kinase (PI3K)/protein kinase B (AKT) pathway, mitogen-activated protein kinase (MAPK) pathway, Janus kinase/signal transducers and activators of transcription (JAK/STAT) pathway, WNT signaling pathway and nuclear factor kappa light-chain enhancer of activated B cells (NF-κB) pathway. The induction of the PI3K/AKT pathway can promote *PD-L1* expression. Increased extrinsic signaling, as well as a decreased level of negative regulators, such as phosphatase and tensin homolog (PTEN), may occur [41,42]. In addition, the data indicate that the inhibition of the MAPK pathway may markedly prevent the expression of *PD-L1* [43,44]. Moreover, the results confirm that the JAK/STAT pathway stimulates *PD-L1* expression in cancers [45,46]. Other studies revealed the presence of WNT activity and *PD-L1* expression crosstalk [47,48]. Additionally, a large body of evidence indicates that the induction of NF-κB signals promotes *PD-L1* expression [49,50]. Actually, *PD-L1* expression is upregulated by different molecular mechanisms, including inflammatory signaling, but also by genetic modifications, oncogenic pathways, microRNA or protein-level regulation [51].

PD-1/PD-L1 inhibit the activity of T cells and prevent effector immune cells from killing cancer cells. PD-1/PD-LI inhibitors suppress the PD-1/PD-L1 pathway to restore the normal immune function of T cells. Commonly administered PD-1/PD-L1 inhibitors in the treatment of cancers include, among others, nivolumab, pembrolizumab, atezolizumab, durvalumab and avelumab [52,53]. However, only a subset of PD-L1-positive patients benefits from PD-1/PD-L1 immune therapies. The immunotherapy resistance mechanisms are as follows: changes in T-cell function or proliferation, alternative immunological checkpoints, tumor cell adaptation and alterations of the tumor microenvironment. Therefore, understanding the mechanisms of PD-L1 regulation is helpful to enhance the treatment effect [51,54,55,56,57].

On the one hand, studies indicate that the PD-L1 protein level in tumor cells is positively correlated with a higher tumor grade in breast cancer patients [58] and in squamous cell carcinoma of vulva patients [59]. In addition, upregulated *PD-1* protein expression in T cells may be correlated with immune evasion in gastric cancer patients [60]. Chemotherapy combined with immunotherapy had better overall survival (OS) in non-small cell lung cancer patients regardless of the *PD-L1* expression level [61]. However, a very high *PD-L1* expression was associated with an OS benefit in patients with advanced non-small cell lung cancer receiving PD-L1 inhibitor monotherapy [62]. A study conducted by Taube et al. highlights a therapeutic opportunity in administering factors inhibiting the PD-1 pathway to patients with metastatic melanoma [63]. On the other hand, the *PD-L1* expression of tumor cells was associated with improved OS in pulmonary squamous cell carcinomas [64]. A similar trend was also observed for patients with colorectal [65] and ovarian [66] cancers, as well as esophageal squamous cell carcinoma [67]. At present, it is not known why *PD-L1* expression is associated with a favorable prognosis in certain cancers and a negative prognosis in others. The prognostic impact of this marker is based on incompletely elucidated mechanisms occurring in the tumor microenvironment. 

In our study, we investigated *PD-1* and *PD-L1* expression patterns in the tissues of HNC patients. The research shows increased mRNA and protein levels of *PD-L1* in tumor tissues obtained from patients with HNC compared to the control group. Moreover, a statistically significant higher mRNA expression of *PD-1* was observed in patients with CRSwNP and allergy compared to CRSwNP alone, whereas a higher mRNA expression of *PD-L1* was observed in smoking patients compared to nonsmoking. Melliante et al. point out that immunotherapy has a higher efficacy in smoking patients. This may be attributed to an increase in the number of genetic mutations, which leads to greater immunogenicity [51]. According to our data, the ages of the HNC patients correlated with the expression of the *PD-L1* gene. There was no correlation between TNM staging and the expression of the *PD-1* and *PD-L1* genes. However, it has been suggested that *PD-L1* mRNA expression may be associated with increased local lymphocytic infiltration and OS [68]. 

Our research supports the role of the PD-1/PD-L1 axis as an immune modulator in patients with HNC. Lyu et al. demonstrated that *PD-L1* expression is upregulated in the tumor tissue of HNCs. *PD-1/PD-L1* expression was significantly associated with radiosensitivity, and high *PD-1* expression was strongly related to HPV/p16-positive HNCs [69]. On the one hand, Lin et al. found that a higher level of PD-L1 in HNC tissues was associated with distant metastases and worse outcomes [70]. Similarly, Moratin et al. concluded that elevated *PD-L1* expression in human oral squamous cell carcinomas was correlated with tumor size, stage, regional metastases and worse OS [71]. On the other hand, Müller et al. found that *PD-L1* expression in tumor tissue is correlated with better OS [72]. Other reports show that higher *PD-1* and *PD-L1* expression was associated with a better outcome with fewer local and distant recurrences, mainly in HPV-positive patients [73,74]. Hong et al. indicate that HPV- and PD-L1-positive patients with tonsillar cancer had a better OS, longer progression-free survival and decreased risk of death [75]. Moreover, it was indicated that the therapeutic response of HNC patients with an elevated expression of *PD-L1* to immunotherapy was better [76]. Chen et al. reported that HNC patients with elevated *PD-L1/PD-1* expression tend to have better OS outcomes and a lower probability of recurrence, providing more evidence for the PD-1-targeted immunotherapy of HNCs [77]. On the contrary, Kim et al. indicated that tumor cell expression of *PD-L1* is not associated with clinical characteristics and the prognosis of HNC patients [78]. Therefore, the current results are still controversial.

According to the hypothesis, the studies showed that the level of expression of *PD-1* and *PD-L1* is significantly higher in both patients with NHC and CRSwNP compared to controls. However, our study’s limitation is the small sample size; therefore, further investigation is needed. Elucidating the role of the PD-1/PD-L1 axis may, in the future, facilitate the implementation of effective treatments for immune-related diseases, such as CRS and HNC [79,80].

## 5. Conclusions

Immune-related diseases are becoming common both in male and female patients. Searching for new potential biomarkers for the early detection of these diseases is extremely important, as it will allow to immediately monitor their development. Our studies showed an increased expression of the *PD-1* and *PD-L1* genes, which may be a potential biomarker of chronic sinusitis with nasal polyps.

## Figures and Tables

**Table 1:** Participants’ characteristics.

Analyzed Trait	I Group (n = 102)	II Group (n = 162)	III Group (n = 40)
Gender:			
Female (n/%)	24 (23.5%)	63 (38.9%)	4 (10.0%)
Male (n/%)	78 (76.5%)	99 (61.1%)	36 (90%)
Age (years) ± SD	52.74 ± 9.77	50.11 ± 11.88	66.45 ± 10.50
Smoking status “Yes” (n/%)	20 (19.6%)	48 (29.6%)	34 (85.0%)
Kennedy scale	N/A		N/A
1	N/A	6	N/A
2	N/A	30	N/A
3	N/A	78	N/A
4	N/A	48	N/A
TNM grading			
T1N0M0 (n/%)	N/A	N/A	20 (50.0%)
T2N0M0 (n/%)	N/A	N/A	8 (20.0%)
T3N0M0 (n/%)	N/A	N/A	12 (30.0%)

**Table 2:** mRNA expression level of *PD-1* and *PD-L1* genes in different subgroups of CRSwNP and HNC patients.

Groups	*PD-1* Expression	*p*-Value	*PD-L1* Expression	*p*-Value
CRSwNP Patients				
Control (n = 102)	2.45 ± 0.22	Ref.	1.87 ± 0.19	Ref.
CRSwNP (n = 77)	5.88 ± 0.58	<0.001	4.31 ± 0.71	<0.001
CRSwNP with allergy (n = 26)	14.95 ± 1.12	<0.001	4.49 ± 0.63	<0.001
CRSwNP with N-ERD (n = 59)	10.53 ± 3.701,22	<0.001	4.11 ± 0.53	<0.001
				
CRSwNP (n = 77)	5.88 ± 0.58	Ref.	4.31 ± 0.71	Ref.
CRSwNP with allergy (n = 26)	14.95 ± 1.12	<0.001	4.49 ± 0.63	0.888
CRSwNP with N-ERD (n = 59)	10.53 ± 3.70	0.051	4.11 ± 0.53	0.831
HNC Patients				
Control (n = 102)	2.45 ± 0.22	Ref.	1.87 ± 0.19	Ref.
Nonsmokers (n = 6)	10.54 ± 1.22	<0.001	5.98 ± 0.87	<0.001
Smokers (n = 34)	11.27 ± 1.01	<0.001	11.33 ± 1.02	<0.001
				
Nonsmokers (n = 6)	10.54 ± 1.22	Ref.	5.98 ± 0.87	Ref.
Smokers (n = 34)	11.27 ± 1.01	0.770	11.33 ± 1.02	0.037

**Table 3:** Multiple linear regression analysis for age and extent of the diseases on the mRNA expression level of the *PD-1* and *PD-L1* genes.

	CRSwNP Patients			HNC Patients (n = 40)		
	B ± SE	β	*p*-Value	B ± SE	β ± SE	*p*-Value
	*PD-1* mRNA Expression Level					
Age	−0.002 ± 0.017	−0.022	0.900	−0.007 ± 0.308	−0.047	0.778
Kennedy scale	−0.511 ± 0.251	−0.360	0.043	N/A	N/A	N/A
TNM scale	N/A	N/A	N/A	0.468 ± 0.308	0.249	0.137
	*PD-L1* mRNA Expression Level					
Age	0.013 ± 0.008	0.276	0.106	0.079 ± 0.015	0.647	<0.001 ***
Kennedy scale	−0.502 ± 0.123	−0.694	<0.001 ***	N/A	N/A	N/A
TNM scale	N/A	N/A	N/A	0.329 ± 0.183	0.225	0.079

B = regression coefficient; SE = standard error; β = standardized regression coefficient. *** *p* ≤ 0.001.

## Data Availability

The data analyzed in the study are available upon request to the authors of the article.
